# Co-Application with Tannic Acid Prevents Transdermal Sensitization to Ovalbumin in Mice

**DOI:** 10.3390/ijms23073933

**Published:** 2022-04-01

**Authors:** Eri Izumi, Nana Tanahashi, Serina Kinugasa, Shota Hidaka, Nobuhiro Zaima, Tatsuya Moriyama

**Affiliations:** 1 Department of Applied Biological Chemistry, Graduate School of Agriculture, Kindai University, 3327-204, Naka-machi, Nara 631-8505, Japan; erizumi.123@gmail.com (E.I.); 7.tanahashi@gmail.com (N.T.); serina.kinugasa0106@gmail.com (S.K.); shotahidaka14@gmail.com (S.H.); zaima@nara.kindai.ac.jp (N.Z.); 2 Agricultural Technology and Innovation Research Institute (ATIRI), Kindai University, 3327-204, Naka-machi, Nara 631-8505, Japan

**Keywords:** allergen, tannic acid, epidermal sensitization, IgE, IgG1, ovalbumin, inhibitory effect, TSLP, TARC

## Abstract

Transdermal sensitization to allergens is of great concern as a sensitization route for food allergies. This skin-mediated invasion and sensitization to allergens is involved in skin barrier breakdown and inflammation, followed by the production of several kinds of cytokines. Cytokines such as thymic stromal lymphopoietin and thymus and activation-regulated chemokine are also involved. In this study, we investigated the suppressive effect of tannic acid (TA) on transdermal sensitization using ovalbumin (OVA), a major egg-white allergen. We also analyzed the mechanisms associated with the inhibitory effects of TA. The results showed that the co-application with TA prevents transdermal sensitization to OVA. As possible mechanisms, its anti-inflammatory and astringent effect on the skin and binding ability with the protein were considered. These results indicate that TA could be applied to cosmetics and lotions, which could suppress the transdermal sensitization to allergens.

## 1. Introduction

Percutaneous sensitization, wherein sensitization is established by the invasion of allergens through the skin rather than oral sensitization, has recently gained attention as a sensitization route for food allergies [1]. This is based on the dual-antigen exposure hypothesis proposed by Lack in 2008, which suggests that allergy is induced by proteins that invade the skin, but that immune tolerance is induced by orally ingested food [2]. It has been reported that allergic reactions by percutaneous sensitization are caused by various foods. In our previous studies, we identified the major percutaneously sensitizing allergen candidates in soybean [3,4], cherry [5], and kiwi [6]. Ovalbumin (OVA) and lactoglobulin were also reported to be percutaneously sensitizing allergens in egg and milk, respectively [7,8]. However, studies on inhibitory compounds against percutaneously sensitizing allergens co-applied on the skin have not been conducted.

We recently found that tannin-containing foods may have little sensitizing potential [9]. Therefore, we speculated that tannin or tannin-related compounds might have an inhibitory effect on percutaneously sensitizing allergens when co-applied on the skin.

**Figure 1:**
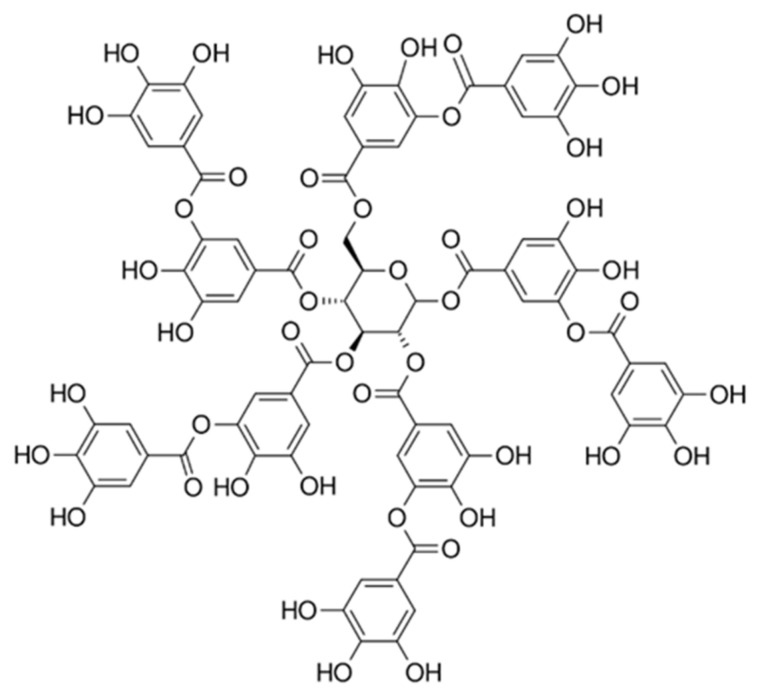
Chemical structure of tannic acid (TA) (C_76_H_52_O_46_).

Tannic acid (TA) (Figure 1) is a naturally occurring plant polyphenol found in nearly all aerial plant tissues. It has been used historically for treating diarrhea, skin burns, and rectal disorders [10]. In addition, tannins have been documented to exhibit antioxidant, antibacterial, antiviral, antiprotozoal, and anti-carcinogenic effects [11,12,13,14,15]. However, the effect of TA or tannins on the transdermal sensitization process of antigen proteins has not yet been investigated.

Therefore, in this study, we used mouse models to determine whether the co-application of TA and OVA to the skin could suppress the transdermal sensitization ability of OVA, which is a major allergen of egg whites. The mechanism underlying the inhibitory mechanism of TA on transdermal sensitization was also investigated.

## 2. Results

### 2.1. Effects of the Topical Application of OVA and TA on Growth and Serum Parameters

**Figure 2:**
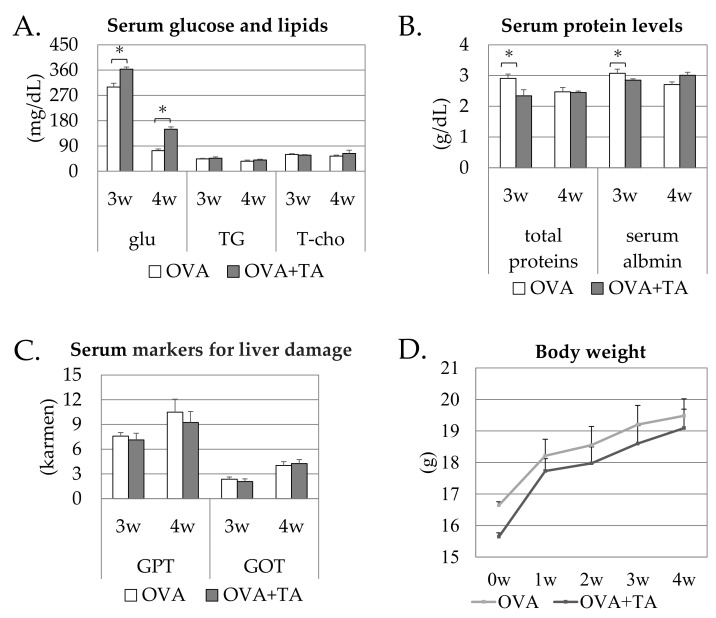
Serum parameters of mice at 3 w and 4 w in OVA and OVA + TA groups. (**A**) Serum glucose, triglyceride, and total cholesterol concentrations. (**B**) total proteins and serum albumin concentrations. (**C**) serum glutamic-pyruvic transaminase (GTP) and glutamic-oxaloacetic transaminase (GOT) activity of each mouse group at 3 w and 4 w. (**D**) Body weight changes of each mouse group from 0 w to 4 w. All data are expressed as mean ± standard deviation; OVA group (*n* = 6), OVA + TA group (*n* = 6). * *p* < 0.05.

As the nutritional conditions of animals are generally well known to be related to allergenic responses, we carefully examined growth and serum parameters in the OVA and OVA + TA groups. As shown in Figure 2, the serum glucose levels at 3 and 4 weeks were significantly higher than those in the control groups (*p* < 0.05). Other serum parameters were comparable and not significantly different. In addition, body weight gradually increased over time, but no statistically significant difference was observed in body weight changes between the groups. The average initial body weights were slightly different between two groups, but the difference was approximately 1 g. This difference was not considered physiologically meaningful; hence, the mice were reared normally, and no differences in growth were observed between groups.

### 2.2. Effect of Co-Application of TA and OVA on OVA-Specific IgE, IgG1 and IgG2a Production

**Figure 3:**
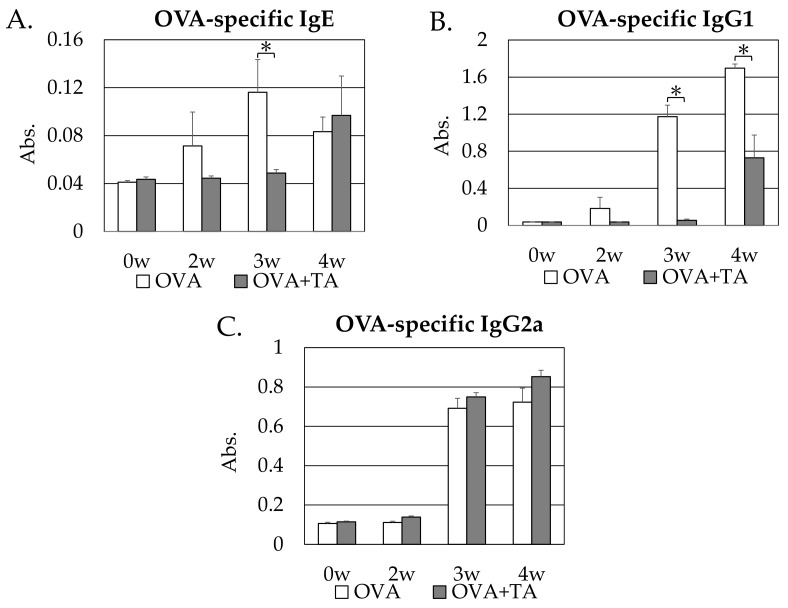
OVA-specific IgE and IgG1 levels of sera in the OVA and OVA + TA groups determined by ELISA. (**A**) OVA-specific IgE, (**B**) OVA-specific IgG1, and (**C**) OVA-specific IgG2a levels in each mouse group at 0 w, 2 w, 3 w, and 4 w. Absorbance data are expressed as mean ± standard deviation; OVA group (*n* = 6), OVA + TA group (*n* = 6). * *p* < 0.05.

Enzyme-linked immunosorbent assay (ELISA) was performed using sera from mice at 0, 2, 3, and 4 weeks upon transdermal sensitization treatment. OVA-specific IgE (Figure 3A), IgG1 (Figure 3B), and IgG2a (Figure 3C) levels were measured, and the levels of the former two antibodies were significantly lower in the OVA + TA group than in the OVA group at 3 weeks (*p* < 0.05). These antibody values were almost the same as those before OVA application (0 weeks). The results indicated that the dermal sensitization to OVA was suppressed on co-application with TA, despite the disruption of the skin barrier by sodium dodecyl sulfate (SDS) application and tape removal. Conversely, differences in OVA-specific IgG2a levels were not statistically significant between the two groups. These results indicated that co-application of TA might not suppress OVA-specific IgG2a production.

### 2.3. Effect of Co-Application of TA and OVA on OVA-Specific IgG1 Production 

**Figure 4:**
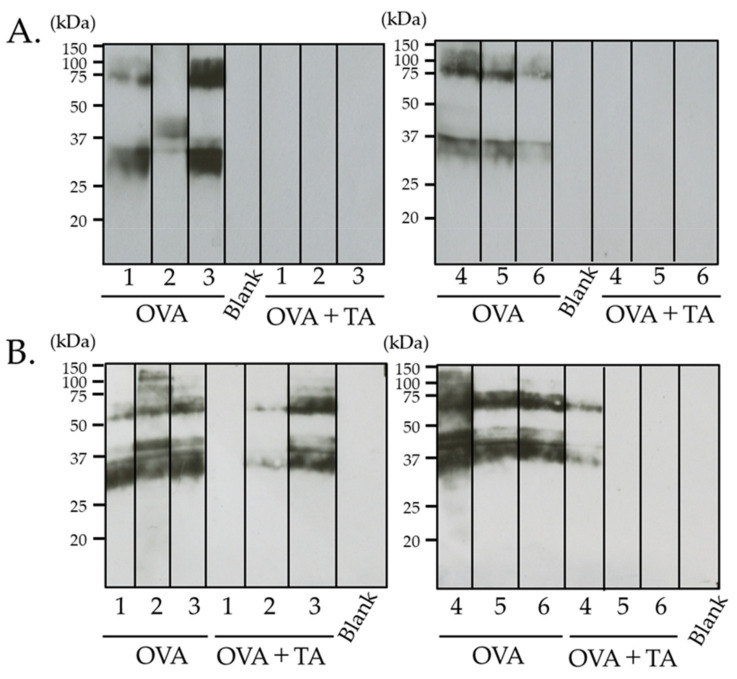
Immunoblotting analysis of IgG1-binding protein bands. Immunoblotting using OVA proteins, individual mice sera at (**A**) 3 w and (**B**) 4 w, and HRP-labeled anti-mouse IgG1 antibody. The numbers indicate the individual mice in the OVA and OVA + TA groups. Molecular mass was expressed in kDa. No specific bands were detected in areas higher or lower than the 15–150 kDa range in sera from mice in both groups (data not shown).

Immunoblotting with OVA (Figure 4) also showed clear IgG1 binding bands in the OVA-treated group. However, no reactive bands were detected in the TA co-application group, suggesting that the production of IgG1 antibodies against the applied antigens containing OVA was extremely low.

The results obtained by immunoblotting supported the ELISA results. In addition, in the OVA-treated group, a band recognized by the IgG1 antibody was also detected near 75–100 kDa and the molecular weight of putative OVA (approximately 40 kDa). Although the identity of this 75–100 kDa protein is unknown, it could be a contamination with ovotransferrin or dimers of OVA, as suggested by the molecular weight.

### 2.4. Effect of Co-Application of TA and OVA on the Thymic Stromal Lymphopoietin and TARC Levels in the Skin

Corneocytes in the skin are damaged during transdermal sensitization, which is hypothesized to trigger the secretion of thymic stromal lymphopoietin (TSLP) [16]. In addition, thymus and activation-regulated chemokine (TARC) is a chemokine produced by epidermal keratinocytes and other cells that causes Th2 cells to migrate to areas of skin involvement. Th2 cells accumulated by the action of TARC exacerbate atopic dermatitis (AD) by increasing allergic responses (e.g., IgE production, eosinophil infiltration, and activation) [17].

**Figure 5:**
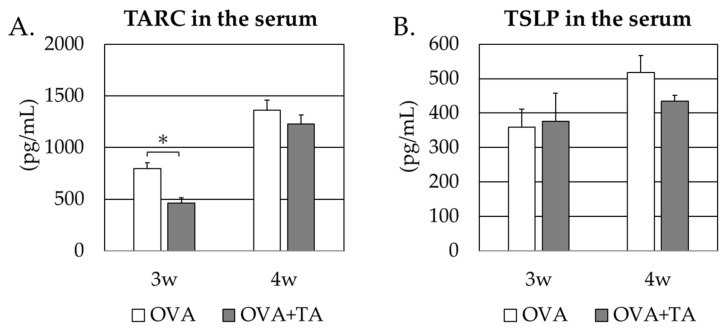
TSLP and TARC levels in the serum from the OVA and OVA + TA groups. (**A**) TARC levels in the serum at 3 w and 4 w. (**B**) TSLP levels in serum at 3 w and 4 w. Data are expressed as means ± standard deviations; OVA group (*n* = 6), OVA + TA group (*n* = 6). * *p* < 0.05.

**Figure 6:**
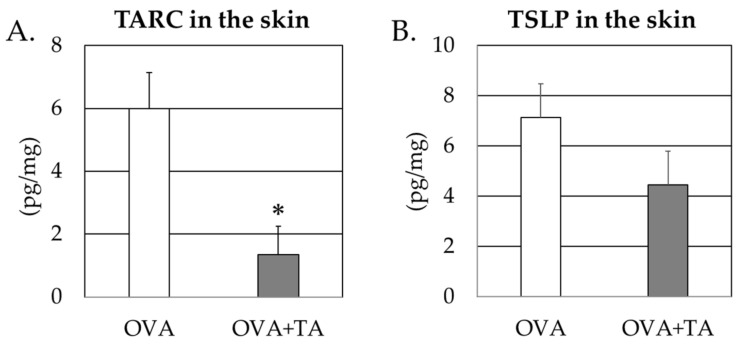
TSLP and TARC levels in the skins from OVA group and OVA + TA group. (**A**) TARC levels in the skin at 4 w. (**B**) TSLP levels in skin at 4 w. Data are expressed as means ± standard deviations; OVA group (*n* = 6), OVA + TA group (*n* = 6). * *p* < 0.05.

TSLP and TARC levels may be linked to Th2-type antibody production and epidermal sensitization to promote allergic responses in the skin, such as inflammatory and Th2 type immune responses. Therefore, commercial ELISA kits were used to determine the serum and skin levels of these cytokines. Figure 5 shows that TSLP levels in the treated skin of the OVA + TA group were lower than those in the OVA-only group. However, there was no significant difference between the groups. In contrast, TARC levels in the skin were significantly lower in the OVA + TA group (*p* < 0.05). There were no significant differences in serum TSLP and TARC concentrations between the two groups (*p* > 0.05) (Figure 6).

### 2.5. Effect of Co-Application of TA and OVA on the Skin

The worsening of allergic symptoms is due to the overproduction of IgE antibodies and the infiltration of inflammatory cells into the skin lesions. 

**Figure 7:**
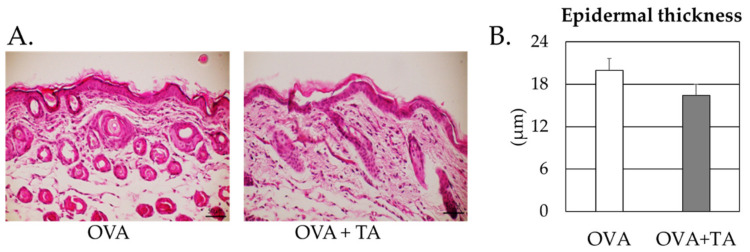
H&E stain results for the skin sections from OVA and OVA + TA groups. (**A**) H & E stain. The scale bars indicate 50 μm. (**B**) epidermal thickness.

We evaluated the effect of TA on the thickness of skin lesions following OVA application using hematoxylin and eosin (H&E) staining. The epidermal thickness in skin sections from the OVA + TA group was lower than that in skin sections from the OVA-only group. However, there was no significant difference between groups (Figure 7).

## 3. Discussion

In this study, as part of a search for natural compounds that can suppress transdermal sensitization responsible for food allergy, we evaluated the suppression of transdermal sensitization by co-applying TA and OVA as an antigen using a mouse model. Previously, we obtained experimental results showing that food proteins that include TA and tannins are less prone to transdermal sensitization [9]. Therefore, in the current study, we verified the inhibitory effect of TA on transdermal sensitization.

TA is primarily used as a fixative for dyes and as a chemical intermediate and reagent in the production of inks, rubber products, imitation horns, and bevels. Oral intake of TA can cause sclerosis of the gastrointestinal mucosa, resulting in reduced nutritional and xenobiotic absorption. It has also been reported that TA could reduce the carcinogenicity of some amine derivatives and polycyclic aromatic hydrocarbons in animal studies [18].

When TA was applied with OVA in the presence of SDS, the effects on growth and serum parameters of mice at 3 and 4 weeks were investigated, and serum glucose levels were significantly higher in the tannin group. For unknown reasons, it is possible that transdermal translocation of TA-derived glucose into the bloodstream may have occurred because one mol of glucose is structurally contained in one mol of TA. Other major mouse serum parameters did not change significantly, and the co-application with TA to the skin was unlikely to have adverse effects on the mice.

OVA was applied to the dorsal surface of the mice in the presence of SDS. OVA-specific IgE antibodies and OVA-specific IgG1 antibodies began to increase at week 2, and both showed significantly higher absorbance values at week 3 than at week 0. In the OVA + TA group, both IgE and IgG1 had significantly lower antibody titers (absorbance) than in the OVA-only group. Especially in the case of IgG1, it was the same level as the value at 0 weeks, and IgG1 antibody production by the transdermal application of OVA was completely suppressed. In the case of IgE, there was no difference between the OVA + TA group and the OVA group at 4 weeks. However, in the case of IgG1, the antibody titer in the OVA + TA group was significantly lower than that in the OVA group even at 4 weeks. Therefore, it was clarified that the co-application with TA suppressed the transdermal sensitization to OVA. However, this effect was attenuated by week 4. These results suggest that the inhibitory effect of TA does not continue for long and that it is strongly effective in delaying transdermal sensitization. The suppressing effect of TA diminished in the 4th week rather than in the 3rd week. It is difficult to speculate on the reasons for this phenomenon from our available data. However, regarding TARC, the inhibitory effect of TA was higher in the 3rd week than in the 4th week; therefore, it is possible that the inhibitory effect on this cytokine might be attenuated in the 4th week.

OVA-specific IgG2a of both groups was significantly increased at week 3 and week 4 compared to week 0, indicating that a Th1-type immune response against applied OVA was activated. However, there were no differences between the two groups, suggesting that the immunosuppressive effect of TA mainly affected Th2-type allergenic responses (IgE and IgG1 production in mice). This result suggested that TA might preferentially suppress Th2-type allergenic responses.

The transdermal sensitization inhibition effect of TA was clearly shown using the murine IgG1 antibody. Similarly to the ELISA findings, at 3 weeks in the OVA group, all mice’s antibody-reactive protein bands were detected. In contrast, in the OVA + TA group, no protein bands were detected. Conversely, at 4 weeks, reactive protein bands were detected from the antibodies of three out of six mice in the OVA + TA group.

In addition, in the OVA-treated group, a band recognized by IgG1 antibody was also detected near 75–100 kDa. However, this unknown protein may have been either an ovotransferrin or a dimer of OVA.

The mechanism of TA’s inhibitory effect on transdermal sensitization by co-application with OVA and the related events were investigated in the present study. Cytokines such as TSLP and TARC, which are associated with skin inflammation and Th2 cell migration, are deeply involved in the invasion of and sensitization to allergens through the skin during transdermal sensitization. Therefore, we first measured the serum levels of these two cytokines and their concentrations in the skin extracts.

TSLP levels in the skin at 4 weeks and the time of dissection were not significantly different in the OVA + TA group compared with the OVA group but tended to be lower. In contrast, TARC levels were significantly lower in the OVA + TA group than in the OVA group. In the case of TSLP, there were no significant differences between the two groups at 3 or 4 weeks in the case of sera, but in the case of TARC, the values were significantly lower in the OVA + TA group than in the OVA group at 3 weeks. These findings also suggest that local inhibition of the production of cytokines involved in dermal sensitization, such as TARC and TSLP, and allergic responses in the skin may be related to the inhibition of transdermal sensitization by TA. Indeed, it has been reported that TAs and quercetin may be therapeutically effective in AD by suppressing the expression of Th2 related cytokines, including TSLP and TARC, in Nc/Nga murine models showing pathologies such as AD [19,20]. In addition, mite-induced AD may be induced via NF-κB signaling, and some reports indicate that TA may inhibit its signaling and inflammation [21]. For AD, bathing agents containing TA have been shown to aid skincare in people with mild to moderate AD itching and skin involvement [22], and the efficacy of TA in preventing inflammation in the skin is promising.

TA inhibits IgE production by inhibiting STAT6 phosphorylation and ɛGT expression induced by IL-4 and IL-13. Therefore, a direct effect on immunocompetent cells may also be involved [23]. TA also decreased the production of inflammatory cytokines, IgE, and histamine in an OVA-induced rhinitis murine model (BALB/c) [24,25].

In addition, the efficacy of TA as a barrier protectant to reduce skin damage has been evaluated through squamometry [26]. This suggests that TA exerts binding or astringent effects on the skin, which may be useful in restoring barrier health.

Finally, because TA has the property of binding to proteins in a non-specific manner, it is also likely that the binding of the antigenic protein and TA may physically prevent the capture of immune cells. This is supported by the findings of Chen et al., which show that TA binds strongly to OVAs [27,28,29].

The binding and protective effects of TA toward these antigenic proteins may also be involved in the inhibition of transdermal sensitization. Among these multiple effects of TA, the most important in transdermal sensitization is unclear. Additionally, these various effects are likely related to the transdermal sensitization control of the allergen. Further studies are required to elucidate these mechanisms.

Recently, epigenetic modifications of gene expression in allergy-related molecules have been shown to affect allergenic responses [30,31]. Therefore, the suppressive effect of TA might be involved in the epigenetic modifications of key molecules. In the future, this point should be investigated.

Patients with AD are known to develop (transdermal) sensitization to various food and environmental allergen components, including egg allergens, which is considered to be the rationale for atopic march [32,33]. Since TA suppressed transdermal sensitization to OVA in the present mouse model, it may have the potential to suppress the transdermal sensitization to various antigens in atopic march. Therefore, a practical implication of this study is the possibility that TA could be applied to cosmetics and lotions that could suppress the transdermal sensitization to allergens and sequentially occurring allergenic disorders in allergic (atopic) march.

Here, we examined the effect of TA only on mice. This point may be a limitation of the study. Further clinical studies are required to elucidate the effects of TA on transdermal sensitization in humans.

## 4. Materials and Methods

### 4.1. Materials

OVA (95% purity) was obtained from Sigma-Aldrich, and TA was obtained from Fujifilm Wako Pure Chemicals (Osaka, Japan). ELISA plates were purchased from Asahi Glass (Tokyo, Japan). Horseradish peroxidase (HRP)-conjugated goat anti-mouse IgG1 antibody was purchased from Bethyl Laboratories (Montgomery, TX, USA). 3,3′,5,5′-tetramethylbenzidine (TMB) peroxidase substrate was purchased from Kirkegaard & Perry Laboratories (KPL, Gaithersburg, MD, USA). CanGet Signal Solutions 1 and 2 were purchased from Toyobo (Osaka, Japan). The PVDF membrane (Immobilon^TM^-P) was purchased from Millipore (Billerica, MA, USA). Enhanced chemiluminescence (ECL) Western blotting substrate and X-ray films (Amersham Hyperfilm^TM^ MP) were purchased from GE Healthcare (Chalfont St. Giles, UK). All other chemicals used in this study had the highest available purity.

### 4.2. Percutaneous Sensitization Treatment

**Figure 8:**
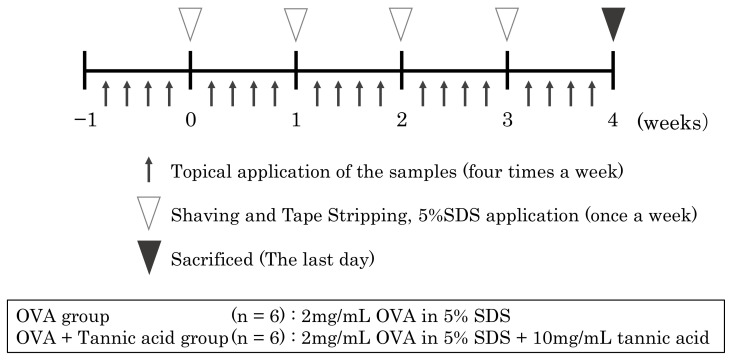
Schema of the percutaneous sensitization protocol in this study. Detailed information is described in the Materials and Methods.

Six-week-old female BALB/c mice were purchased from Japan SLC Inc. (Shizuoka, Japan) and used for percutaneous sensitization treatment, as shown in Figure 8. After acclimatization for 1 week, they were divided into two groups (OVA group, OVA + TA group), and samples were applied four times a week for 4 weeks. Shaving and tape stripping were performed once per week under anesthesia. Once a week, the mice were anesthetized, their backs were shaved, and tape stripping (10 times) using adhesive tape and 5% SDS application were performed to damage the stratum corneum in the skin.

A mixture of midazolam (Astellas Pharma, Tokyo, Japan), butorphanol (Meiji Pharmaceutical, Tokyo, Japan), and medetomidine (Nippon Zenyaku Kogyo, Fukushima, Japan) was used for anesthesia. The treatment was performed weekly. Transdermal sensitization was induced by applying the respective samples to the skin after shaving. The OVA group received 2 mg/mL of OVA in 5% SDS, and the OVA + TA group received 2 mg/mL OVA and 1% (10 mg/mL) TA in 5% SDS. Four times a week for a total of 4 weeks, 25 μL of each sample was applied to the epidermis using a micropipette. At 4 weeks, all mice were anesthetized by intraperitoneal injection of sodium pentobarbital salt. The mice were then sacrificed by cervical dislocation. The treated skin areas were recovered and stored at −80 ℃ until analysis. The mice were fed commercial chow without OVA (MF, Oriental Yeast, Tokyo, Japan), and water was provided ad libitum. All animal experiments were approved by the Kindai University Animal Experiment Commission (approval no. KAAG-26-004).

### 4.3. Enzyme-Linked Immunosorbent Assay

ELISA was used to determine the binding of mouse serum IgG1, IgG2a or IgE to OVA. OVA dissolved in phosphate-buffered saline (PBS) (protein concentration 20 µg/mL) was immobilized at 4 °C overnight at 50 µL/well. After washing, 100 μL of a 1% BSA solution (1% BSA/PBST) dissolved in 0.1% Tween 20-containing PBS (PBST) was added to each well of the ELISA plates and blocked for 1 h. Then, three washes with 100 μL of PBST and 1:100 (for IgE and IgG1 measurements) or 1:50 (for IgG2a measurement) dilutions of mouse sera in 1% BSA/PBST were added to the wells and incubated for 1 h at 37 °C. After washing the wells with 100 μL of PBST five times, 1:8000-fold dilution of rat monoclonal 23G3 anti-mouse IgE epsilon chain HRP-conjugated secondary antibodies (Abcam, Cambridge, UK) in 1% BSA/PBST, and 1:50,000 dilutions of HRP-conjugated anti-mouse IgG1 antibody (Bethyl Laboratories, USA) in 1% BSA/PBST, and 1:500 dilutions of goat anti-mouse IgG2a-HRP-conjugated secondary antibodies (SouthernBiotech, Birmingham, USA) in 1% BSA/PBST were added to the wells and incubated for 1 h at 37 °C. After five washes with 100 μL of PBST, the bound secondary antibodies were detected by reaction with 50 μL of a TMB peroxidase substrate. Finally, the reaction was stopped by adding 50 µL of 1 M phosphoric acid. Absorbance was measured using a plate reader (Wallac ARVO SX 1420 Multilabel Counter; PerkinElmer, Waltham, MA, USA) at 450 nm.

### 4.4. Electrophoresis and Immunoblotting

OVA was subjected to SDS-PAGE [34]. Proteins in the 12.5% gel were stained with Coomassie brilliant blue (CBB) (CBB R-350, GE Healthcare) to visualize the total protein patterns. Immunoblotting analysis was conducted by transferring the SDS-PAGE gel to an Immobilon-P^TM^ PVDF membrane (Millipore) using a semi-dry blotting method [35]. The membrane was immersed in 10 mM PBS (pH 7.5) containing 0.1% Tween-20 (PBST) and 5% skim milk for blocking. The membrane was incubated overnight with mouse sera (1:100 dilution) at 4 °C. After washing with PBST three times for 10 min, the protein surface of the membrane was reacted with 1:50,000 dilutions of HRP-conjugated anti-mouse IgG1 antibody for 1 h. After washing the membranes four times with PBST for 10 min, the bound primary antibodies were detected using HRP-conjugated goat anti-mouse IgG1 and an ECL^TM^ Western blotting kit (GE Healthcare). The resultant chemiluminescent signals were detected on an X-ray film (Hyperfilm^TM^ MP, GE Healthcare).

### 4.5. Determination of Serum or Skin TSLP and TARC Concentrations in Mice

The collected mouse skin sections were homogenized in radioimmunoprecipitation assay (RIPA) buffer and centrifuged. The supernatant was used for the determination of TSLP and TARC levels using a commercial ELISA kit (DuoSet Kit, R&D Systems, Minneapolis, MN, USA). The protein concentrations were also determined by the Bradford method [36], and the TSLP and TARC levels were expressed as ng/mg skin proteins.

### 4.6. H&E Stain

After the mice were sacrificed, the dorsal skin was removed, fixed in 4% paraformaldehyde (Nacalai Tesque), embedded in paraffin (Sakura Finetek Japan Co., Ltd., Tokyo, Japan), and sectioned (10-µm thick sections). The sections were then stained with H&E solution (Sakura Finetek Japan Co., Ltd., Tokyo, Japan). The epidermal thickness was measured using ImageJ software (National Institutes of Health, Bethesda, MD, USA). 

### 4.7. Statistical Analysis

Student’s t-test was used to compare the two groups. Statistical analysis was performed using StatView 5.0, and statistical significance was set at *p* < 0.05.

## Data Availability

The data presented in this study are available upon request from the corresponding author.
